# Prevalence of precancerous cervical lesions and associated factors among HIV-positive women in Zewditu Memorial Hospital, Addis Ababa, Ethiopia

**DOI:** 10.3389/fonc.2025.1545829

**Published:** 2025-06-11

**Authors:** Abebech Haile Dinberia, Habtamu Molla Ayele, Meaza Zeleke Wodajo

**Affiliations:** ^1^ Department of Public Health, Yekatit 12 Hospital Medical College, Addis Ababa, Ethiopia; ^2^ Maternal and Child Health Directorate, Federal Ministry of Health, Addis Ababa, Ethiopia; ^3^ Non-Communicable Diseases Research Directorate (NCDRD), Armauer Hansen Research Institute (AHRI), Addis Ababa, Ethiopia

**Keywords:** precancerous cervical lesions, cervical cancer, HIV, HPV, Addis Ababa, Ethiopia

## Abstract

**Background:**

Cervical cancer is one of the major causes of morbidity and mortality among women in low- and middle-income countries. This problem is more severe in developing countries than in developed countries. This study was designed to identify the prevalence and determinants of precancerous cervical lesions among HIV-positive women in the Zewditu Memorial Hospital, Addis Ababa, Ethiopia.

**Objective:**

This study aimed to assess the prevalence of precancerous cervical lesions and associated factors among HIV-positive women in Zewditu Memorial Hospital, Addis Ababa, Ethiopia.

**Methods:**

An institution-based, cross-sectional, quantitative study was conducted. The sample size for the study was 361, which was calculated using a single population proportion method, and the final study participants were HIV-positive clients who were eligible for cervical cancer screening and selected using a simple random sampling method. Data were collected from each study participant using an interviewer-administered questionnaire. Data were then entered into Epi Info version 7, cleaned, and analyzed using SPSS version 25. Bivariable and multivariable logistic regression analyses were used to examine the relationship or statistical association between the independent and dependent variables, and odds ratios with 95% confidence intervals were computed.

**Results:**

A total of 361 women who had follow-ups at an antiretroviral therapy clinic were included in the study. The prevalence of precancerous cervical lesions was 16.6%. Having a history of sexually transmitted disease [adjusted odds ratio (AOR) = 4.88, 95% confidence interval (CI): 1.66–14.36, p-value = 0.004], a history of smoking (AOR = 9.35, 95% CI: 3.15–27.75, p-value = 0.000), and abortion (AOR = 3.23, 95% CI: 1.58–6.58, p-value = 0.001) were significantly associated with precancerous cervical lesions.

**Conclusion and recommendations:**

The prevalence of precancerous cervical lesions in this study was high. A history of abortion, smoking, and sexually transmitted infections were strongly associated with precancerous cervical lesions among women with human immunodeficiency virus. Hence, it is important to emphasize the importance of preventing sexually transmitted infections and repeated abortions, as well as discouraging cigarette smoking.

## Introduction

Cervical cancer is a sexually transmitted disease (STD) that results from infection with oncogenic types of human papillomavirus (HPV) (1) and is responsible for 99% of cases. The first sexual encounter occurring at a young age (16 years), having multiple sexual partners, smoking, and high parity are all associated with HPV infection and cervical cancer (2). Globally, cervical cancer is the fourth most common cancer among women, with over 570,000 new cases and 311,000 deaths due to cervical cancer occurring worldwide in 2018 (3). Among cervical cancer mortalities, nine out of 10 (90%) occurred in low- and middle-income countries (LMICs). Cervical cancer is one of the leading causes of morbidity and death among women in low- and middle-income countries, where inadequate access to screening and treatment services accounts for more than 85% of the worldwide burden and deaths (3, 4). It is the second most common cancer in Africa, with 80,400 women being diagnosed annually. According to recent data, 50,300 people die of cancer annually, making it the most common cause of death. The incidence and death rates are five times higher in East Africa, Ethiopia’s home region, and West Africa than in North Africa (5).

Cervical cancer (CC) is an AIDS-defining disease that most commonly affects women living with HIV (WLHIV) (6). The guidelines for cervical cancer prevention policies are provided by the World Health Organization (WHO), including the requirements for WLHIV. Comprehensive global data on the incidence and progression of cervical lesions in women with HIV are crucial for understanding the natural progression of cervical neoplasia and for shaping screening policies. Women who are HIV-positive experience cervical lesions at a rate three times higher than those who are HIV-negative (7). According to the WHO, in regions where HIV is prevalent, 15%–20% of the target population may test positive for precancerous cervical lesions during screening. In HIV-positive women, cervical cancer may develop into an invasive cancer and is estimated to kill approximately 43,000 women by 2030, with more than 98% of deaths expected to occur in developing countries (6). The severity of the anomalies was determined by the precancerous cervical lesion grades, with mild, moderate, and severe labeling. Moderate and severe high-grade cervical intraepithelial neoplasm (CIN) grades are essential for the progression of cervical precancerous lesions to invasive cervical cancer (8). Cervical cancer can be tackled by precancerous lesion screening using visual inspection with acetic acid, followed by suitable treatments. Progression to invasive cervical cancer may take more than 20 years after the first human papillomavirus infection appears, with low-grade cervical dysplasia (CIN1) growing slowly and progressing more quickly to high-grade cervical dysplasia (CIN2 or CIN3) (8).

Cervical cancer is prevented by vaccination against human papillomavirus and timely cervical cancer screening, which aids in the early detection and treatment of precancerous lesions (9). In Sub-Saharan Africa, cervical cancer is a concurrent epidemic with HIV and ranks as the second leading cause of cancer-related fatalities among women (3). However, health facility managers, healthcare providers, and community members have limited awareness of the importance of cervical cancer prevention, leading to the low prioritization of the service. Screening often lacks dedicated space, is interrupted due to staff multitasking, and suffers from a shortage of trained personnel. Weak leadership commitment to demand creation further hinders service uptake. Without strong advocacy and awareness, few women have access to cervical cancer screening services. Despite the absence of a national cancer registry in Ethiopia, analyses of past biopsy data have shown that cervical cancer is the most common cancer among women in the country, with breast cancer being the second most common (9). Women and community members should be informed of the risks of cervical cancer, including its potential to cause morbidity and mortality. Therefore, this study aimed to assess the prevalence of precancerous cervical lesions and associated factors among HIV-positive women in Zewditu Memorial Hospital Addis Ababa, Ethiopia, in 2022.

## Materials and methods

### Study area and period

The study was conducted at Zewditu Memorial Hospital. The hospital is located in the Kirkos Sub-city of Addis Ababa City Administration. Zewditu Memorial Hospital is a pioneer in HIV treatment in Ethiopia. It has all specialties, including dermatology, maternal health services such as delivery, adult and pediatric antiretroviral therapy (ART), plastic surgery, and ophthalmology. It serves more than one million people in the city. The hospital is administered by the Addis Ababa Health Bureau, which provides direction for cervical cancer counseling, screening, and treatment for all HIV-positive clients aged 15–49 years. This study was conducted from May 20 to July 20, 2022. The study population was female people living with HIV/AIDS (PLWHA) aged 15–49 years who– attended the ART clinic at Zewditu Memorial Hospital, Addis Ababa, Ethiopia. The total number of ART clients in this clinic was 7,550, and the number of clients eligible for cervical cancer screening was 2,400.

### Study design

A hospital-based, cross-sectional, quantitative study was conducted.

#### Target population

The target population was HIV-positive women who were followed up at Zewditu Memorial Hospital, Addis Ababa, Ethiopia.

#### Source population

The source population of the study was all HIV-positive women aged 15–49– years who were receiving follow-up care at Zewditu Hospital in Addis Ababa, Ethiopia.

#### Study participants

The study participants were HIV-positive women who were screened using acetic acid for the detection of cervical cancer lesions at Zewditu Memorial Hospital Addis Ababa, Ethiopia.

### Inclusion criteria

All eligible (15–49-year-old– female) HIV-positive clients who had follow-ups at the ART clinic, were screened for cervical cancer lesions, and volunteered for the study were included in the study.

### Exclusion criteria

HIV-positive women who were screened for cervical cancer but did not receive their results or had unknown results were excluded from the study.

### Sample size determination

For the first objective, the sample size was determined using a single population proportion formula. According to the study conducted in Southern Ethiopia, cross-sectional study research was conducted on the prevalence of precancerous cervical lesions among HIV-positive women, and 99 (22.1%) of 448 participants were found to be positive for precancerous cervical lesions (5).

With a Z_a/2_ value of 1.96 and a marginal error of 5%, the sample size was calculated as follows:


$$\frac{\text{n}={\left(\text{Z}\text{a}{/}_{2}\right)}^{2}\text{P}(1-\text{P})}{{\text{d}}^{2}}$$

$$\frac{\text{n}=({\left(1.96\right)}^{2}0.78(1-0.78))}{{\left(0.5\right)}^{2}}$$

where n = 289 with a 10% non-response rate, d is the acceptable margin of error (precision of measurement), z is the standard variant (1.96), and p is the prevalence of precancerous lesions among HIV-positive clients.

To determine the sample size for the second objective of the study, various factors significantly associated with the precancerous cervical lesions were considered with the following assumptions: 95% confidence interval, power 80%, adjusted odds ratio (AOR) for each factor, and 10% non-respondents using the Epi Info version 7 software (**Table 1**).

**Table 1:** Sample size determination for the second specific objective (10).

Independent variables (factors)	Assumptions	Sample size	10% non-respondent	Total sample size
	95% confidence level, power = 80%, case-to-control ratio = 1:1			
Lifetime history of STD	AOR = 2.30 p = 34.2%	206	21	227
Current HAART use	AOR = 0.52 p = 64.9%	328	33	361
Lifetime number of sexual partners	AOR = 0.33 p = 26.96%	208	21	229

STD, sexually transmitted disease; HAART, highly active antiretroviral therapy; AOR, adjusted odd ratio.

After calculating the required sample size for the selected variables, the maximum sample size was determined by computing the sample size for both objectives. Accordingly, the largest sample size used in this study was 361 from the second objective.

### Sampling technique and procedures

**Figure 1:**
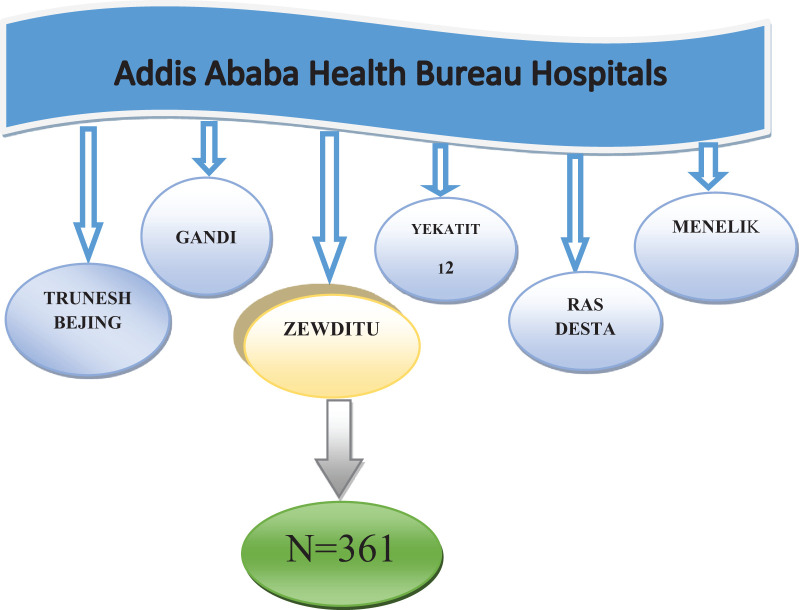
Sampling technique and procedures for determining the prevalence of precancerous cervical lesions and associated factors among HIV-positive women aged 15–49– years in Zewditu Memorial Hospital, Addis Ababa, Ethiopia.

Within the Addis Ababa City Administration, there are six hospitals that offer screening and treatment services for precancerous cervical lesions. Of the six hospitals, one was chosen through a simple random sampling technique. The study population consisted of HIV-positive women who underwent visual inspection with acetic acid for cervical precancerous lesion screening. Women who met the inclusion criteria were consecutively included in the selected 361 patients based on the sample size estimate in the selected hospital (**Figure 1**).

### Data collection procedures

A structured English version was translated into Amharic, and pretested semi-structured questionnaires were used to collect the data. Questionnaires were prepared by reviewing works of literature (5, 11). It has three major components: sociodemographic characteristics, sexual and reproductive history of women with HIV/AIDS, and lifestyle of clients. Data were collected by two health officers and one supervisor with an MSc in public health with previous experience in data collection and supervision. Data were also collected from selected clients at one hospital using a semi-structured interview questionnaire. Before data collection, a 1-day training was provided to both data collectors and supervisors by the principal investigator. The data were collected using interviewer-administered questionnaires. Prior to enrollment in the study, participants provided their informed consent after being thoroughly informed about the study’s objectives, methods, and possible risks and benefits. Participation was voluntary, and confidentiality was maintained by anonymizing the data and implementing restricted access. During the interviews, participants were encouraged to respond openly, and any unclear or confusing questions were clarified by the data collectors. Trained data collectors recorded responses accurately and consistently to ensure the quality and reliability of the data. Written consent was obtained from clients younger than 18 years and signed by their parents.

### Variables

#### Dependent variables

• Prevalence of precancerous cervical lesions.

#### Independent variables

- Age.
- Marital status.
- Religion.
- Number of parities.
- History of smoking.
- History of abortion.
- Family history of cervical cancer.
- Early birth.
- History of alcohol consumption.
- History of pelvic infection.
- Early age sex (less than 18 years).
- Partner history of sexually transmitted infection (STI).
- History of STI.
- Number of partners.
- Highly active antiretroviral therapy (HAART) use.
- Low CD4 count.
- Duration of ART intake.

#### Operational definitions

##### Precancerous cervical lesion

An intraepithelial lesion is an abnormality in the cells of the female cervix that can eventually develop into cervical cancer (12).

##### Cervix

This is the lower narrow end of the uterus that forms between the uterus and vagina (cancer affecting the tip of the cervix is found outside the uterus).

##### VIA

Visual inspection with acetic acid (VIA) is a precancerous cervical screening method using 5% acetic acid. Eligible clients were HIV-positive women aged 15–49 years,– and they were followed up in the ART clinic and tested for cervical cancer.

### Data analysis procedure

Data were entered using Epi Info version 7 and exported to SPSS version 25 for cleaning and further analysis. Descriptive statistics were summarized to describe the quantitative data and percentages for categorical data. The findings obtained from this study are presented in tables and charts, based on the characteristics of the data. Both bivariable and multivariable logistic regression analyses were performed, and the model fitness was checked. The AOR with a respective 95% confidence interval (CI) was used, and a p-value < 0.05 was considered statistically significant. The results of the analysis are summarized and presented in the narratives, along with the quantitative results.

### Data quality management

Prior to data collection, a 1-day training was provided to the data collectors and supervisors on the study instrument and data collection procedure. Pretesting of the questionnaire was conducted 2 weeks before data collection using 5% of the samples in Menelik Comprehensive Specialized Hospital to check for consistency, sequence, clarity, and time required to complete the entire questionnaire. Further modifications were made based on the results of the pilot study. Daily supervision of each data collector was conducted by the supervisor and principal investigator to ensure that the data collection tools were used and completed, and the questionnaires were checked for completeness, legibility, and consistency.

## Result

### Sociodemographic characteristics of the patients

A total of 361 clients aged 15–49– years were interviewed using semi-structured questionnaires with a response rate of 100%. Among the study participants, 12 (3.3%) were aged less than 25 years, and 200 (55.4) were older than 36 years, with a mean age of 37.9, a median age of 38 years, a standard deviation of 6 years, and minimum and maximum ages of 22 and 49 years, respectively. Forty-four (12.2%) of the participants were illiterate, while 226 (62.6%) and 91 (25.2%) attended primary and secondary schools, respectively. Of the total respondents, 230 (63.7%) were married, 6 (1.7%) were single, 95 (26.3%) were divorced, and the rest were widowed and separated; 132 (36.6%) of the respondents were self-employed (merchant), followed by 95 (26.3) housewives, 67 (18.6%) governmental and non-governmental employees, and 67 (18.6%) others like sex workers (**Table 2**).

**Table 2:** Sociodemographic characteristics of HIV-positive women aged 15–49 years in Zewditu Memorial Hospital, Addis Ababa, Ethiopia 2022 (N = 361).

Categories of variables	Category	Frequency	Percent
Age (in years)	<25	12	3.3
	25–30	26	7.2
	31–36	123	34.1
	>36	200	55.4
Marital status	Married	230	63.7
	Single	6	1.7
	Widowed	19	5.3
	Divorced	95	26.3
	Separated	11	3
Occupation	Employed (governmental and non-governmental)	67	18.6
	Housewife	95	26.3
	Self-employed (merchant)	132	36.6
	Others*	67	18.6
Religion	Orthodox	127	35.2
	Muslim	158	43.8
	Protestant	60	16.6
	Others**	16	4.4
Educational status	Illiterate	44	12.2
	Primary education	226	62.6
	Secondary and above	91	25.2

*Others—sex workers and daily laborers.

**Others—Catholic and Jehovah’s Witnesses.

### Reproductive- and behavioral-related factors

Of the study respondents, 28 (7.8%) had a history of smoking. One hundred fifty-three (42.4%) participants started their first sexual intercourse at the age of less than 18 years, and the rest started at 18 years of age and above. Among the respondents, 106 (29.4%) had a history of STI, and 95 (26.3%) had partners with a history of STI. Of the study participants, 353 (97.7%) received HIV care and treatment. One hundred seventy (47.1%) respondents had more than two partners. Three hundred twenty-three (89.5%) participants had given birth to more than two children, and 26 (7.1%) had no history of pelvic infection. Of the total respondents, 304 (84.2%) used contraception methods, and of the total respondents, 18 (5%) had a family history of cervical cancer. Of the total study participants, 329 (91.1%) had no history of alcohol consumption (**Table 3**).

**Table 3:** Reproductive and behavioral factors among HIV-infected women aged 15–49– years in Zewditu Memorial Hospital, Addis Ababa, Ethiopia, 2022 (N = 361).

Categories of variables	Category	Frequency	Percent
Age at first sexual intercourse	<18 years	153	42.4
	≥18 years	208	57.6
Age at first birth	<18 years	38	10.5
	≥18 years	323	89.5
Parity	<2	233	64.5
	≥2	128	35.5
Lifetime number of sexual partners	≤2 partners	191	52.9
	>2 partners	170	47.1
Ever history of STI	Yes	106	29.4
	No	255	70.6
Partner history of STI	Yes	95	26.3
	No	266	73.7
Ever history of genital lesion	Yes	24	6.6
	No	337	93.4
Ever history of pelvic infection	Yes	341	92.9
	No	26	7.1
Family history of cervical cancer	Yes	18	5
	No	343	95
History of abortion	Yes	128	35.5
	No	233	64.5
Ever use of contraceptive	Yes	304	84.2
	No	57	15.8
Use of condom	Yes	16	4.4
	No	345	95
History of smoking	Yes	28	7.8
	No	333	92.2
History of consumption	Yes	32	8.9
	No	329	91

STI, sexually transmitted infection.

### Immunological characteristics of HIV-infected women

Almost all of the study participants (99.1%) were on HAART, and the study participants had CD4 counts greater than 200 cells/mm^3^ after the use of HAART, accounting for 210 (84%), and less than or equal to 200 cells/mm^3^ before the use of HAART, accounting for 280 (77.5) (**Table 4**).

**Table 4:** Immunological characteristics of HIV-infected women in Zewditu Memorial Hospital Addis Ababa, Ethiopia, July 2022 (N = 361).

Categories of variables	Category	Frequency	Percent
Current use of HAART	Yes	358	99.1
	No	3	0.9
>200 CD4 cells/mm^3^ count after use of HAART (N = 250)	Yes	210	84
	No	40	16
≤200 CD4 cells/mm^3^ count before use of HAART (N = 361)	Yes	280	77.5
	No	81	22.4

HAART, highly active antiretroviral therapy.

### Prevalence of precancerous cervical lesion among HIV-infected women in Zewditu Memorial Hospital, Addis Ababa, Ethiopia

**Figure 2:**
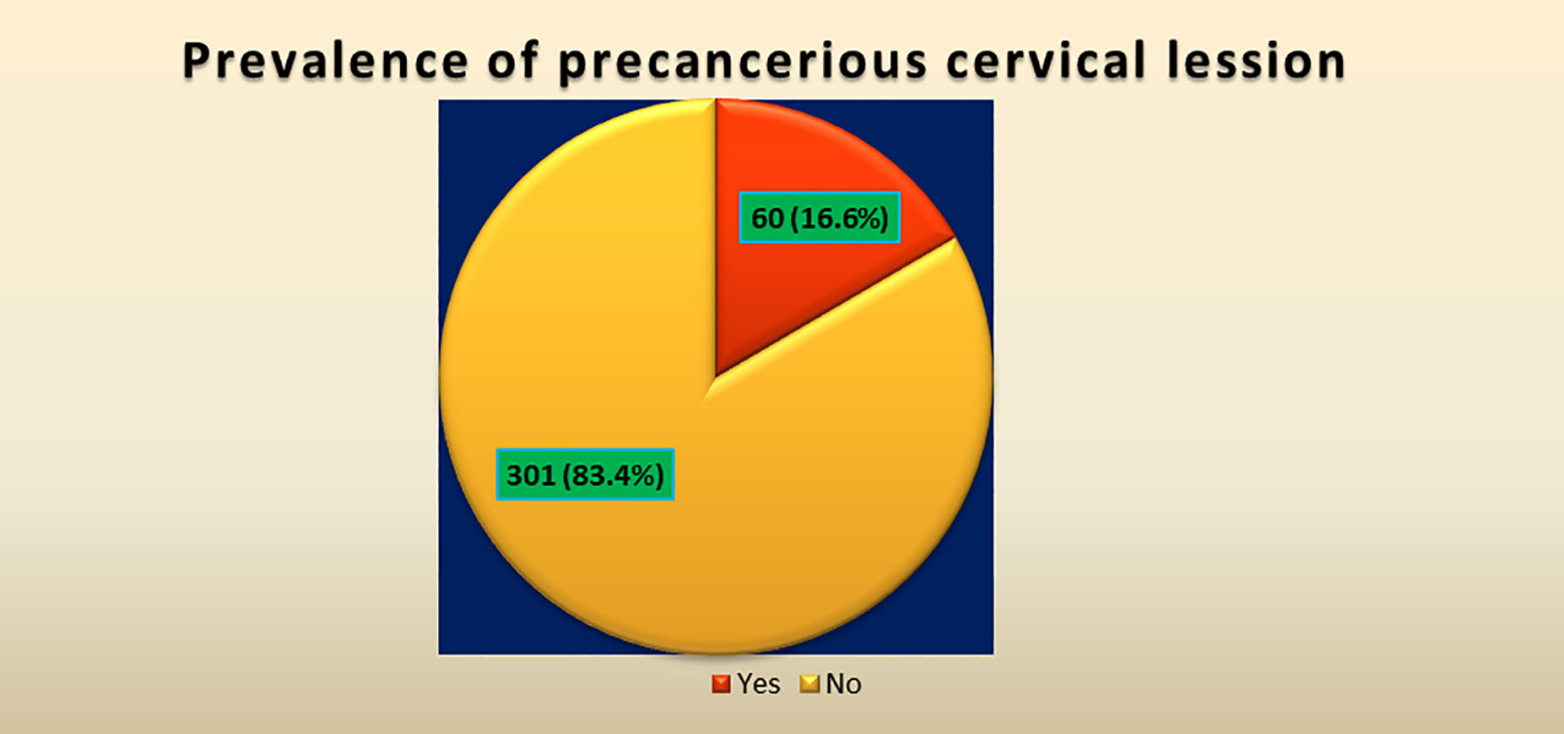
Prevalence of precancerous cervical cancer lesion at Zewditu Memorial Hospital, Addis Ababa, Ethiopia 2022.

Of the total respondents (361), 60 (16.6%) tested positive for precancerous cervical lesions, and the rest had negative test results (**Figure 2**).

### Bivariable and multivariable logistic regression analyses

As described in **Table 5**, both bivariable and multivariable logistic regression analyses were performed to identify independent factors of precancerous cervical lesions among HIV-positive patients. Variables that were significantly associated with the outcome variable (with a p-value less than 0.25) were entered into the multivariable logistic regression analysis to control for the effect of potential confounders. In this study, the variables found to be significantly associated with the outcome variables were history of STI, history of smoking, and history of abortion.

**Table 5:** Bivariable and multivariable analyses of factors associated with sociodemographic, reproductive, and clinical health with precancerous cervical lesion in Zewditu Memorial Hospital Ethiopia, 2022 (N = 361).

Variable	Precancerous cervical lesion			COR (95% CI)	AOR (95% CI)	p-Value
	Yes (%)		No (%)			
History of STI	Yes	41 (39)	65 (61)	0.12 (0.06–0.23)	**4.88 (1.66−14.36)**	0.004*
	No	19 (7)	236 (93)		1	
Partner history of STI	Yes	34 (57)	26 (43)	1.19 (0.109−0.34)	0.92 (0.299−2.855)	0.890
	No	61 (20)	240 (80)		1	
History of alcohol consumption	Yes	11 (18)	49 (82)	0.33 (0.15−0.73)	0.63 (0.192−2.068)	0.446
	No	21 (7)	280 (93)		1	
History of abortion	Yes	36 (28	92 (72)	0.29 (0.16−0.52)	**3.23 (1.58−6.58)**	0.001*
	No	24 (10)	209 (90)		1	
Age at first sexual intercourse	<18 years	34 (22)	119 (88)	0.50 (0.28−0.87)	0.53 (0.222−1.303)	0.170
	≥18 years	26 (13)	182 (87)		1	
History of smoking	Yes	21 (75)	7 (25)	0.04 (0.01−0.11)	**9.35 (3.15−27.75)**	0.000*
	No	39 (12)	294 (88)		1	
Current use of HAART	Yes	57 (95)	3 (5)	3.11 (0.72−13.4)	0.43 (0.970−4.835)	0.436
	No	296 (98)	5 (2)		1	
CD4 less than 200 mm^3^	Yes	24 (40)	36 (60)	0.28 (0.15−0.51)	1.63 (0.676−3.787)	0.284
	No	48 (16)	253 (84)		1	
Ever history of genital lesion	Yes	11 (18)	49 (82)	0.20 (0.08−0.47)	2.00 (0.646−7.4.6)	0.209
	No	13 (4)	288 (96)		1	

COR, crude odds ratio; AOR, adjusted odds ratio; STI, sexually transmitted infection.

^*^Statistically significant at p-value < 0.05.

The study showed that those having a history of smoking were nine times more likely than non-smoking HIV-positive women to develop precancerous cervical lesions (AOR = 9.35, 95% CI: 3.15–27.75, p-value = 0.000), those who had a history of abortion were three times more likely to develop precancerous cervical lesions than their counterparts (AOR = 3.23, 95% CI: 1.58–6.58, p-value = 0.001), and participants with a history of STI were 4.8 times more likely to develop precancerous cervical lesions among HIV-positive women than non-infected women (AOR = 4.88, 95% CI: 1.66–14.36, p = 0.004); these were all independent factors of precancerous cervical lesions (**Table 5**).

## Discussion

Cervical cancer is the top public health challenge in women in Sub-Saharan Africa, accounting for 22.2% of all cancers, and is also the main cause of mortality in women. Precancerous cervical lesions are more common in women with HIV infection than in those without (5). This study aimed to determine the prevalence of precancerous cervical lesions and associated factors among HIV-infected women, which would help in preventing transmutations, and how to increase screening and treatment rates by creating awareness about the severity of the disease at the community level.

This study aimed to assess the prevalence and associated factors of precancerous cervical lesions among HIV-positive women in Zewditu Memorial Hospital, Addis Ababa, Ethiopia. The overall prevalence of precancerous cervical lesions among HIV-positive women aged 15–49 years was 16.6%. The result of this study is almost in line with the results of studies conducted in Southwest Ethiopia (18.7%) (5). In Debre Markos Referral Hospital, Ethiopia, a significantly higher prevalence of epithelial cell disease (17.8%) was detected among HIV-positive women (13). The similarities across these studies may be attributable to the participants sharing the same characteristics, including their status as HIV-positive women and potential behavioral and sexual risk factors.

The findings of this study revealed that only 18 (5%) patients had a family history of cervical cancer, whereas 343 (95%) did not. A similar study in the Amhara region showed that (1.4%) of clients had a family history of cervical cancer; therefore, this study indicates that the majority of cervical lesions are not transferred from the family and are not hereditary. This study’s results showed that 47.1% of the clients had more than two sexual partners and that 42.4% of participants had first sex contact at the age of less than 18 years. A similar study in the Amhara region indicated that more than two sexual partners (58.6%) started their first sex at the age of less than 18 years (71.3%) (11).

A systematic review and meta-analysis in Sub-Saharan Africa indicated that having more than two lifetime sexual partners showed an association with precancerous cervical lesions, and another study in the Amhara region indicated that, in a hospital-based, cross-sectional study, 88.9% of participants who had one sexual partner did not develop precancerous cervical lesions compared to those with multiple sexual partners (4). All the above studies indicated that sexual behavior is one of the main causes of precancerous cervical lesions due to the spread of HPV (14); however, one cross-sectional study conducted in Southwest Ethiopia showed that having one lifetime sexual partner was significantly associated with precancerous cervical lesions, and this outcome is completely different from that of other studies.

In this study, a history of STI, abortion, and smoking were indicated as problems and were significantly associated with precancerous cervical lesions, and they had the same result from different findings in different areas. In this study, a history of STD was significantly associated with precancerous cervical lesions in women. Participants with a history of STI were 4.8 times more likely to develop precancerous cervical lesions among HIV-positive women than among non-infected women. A similar cross-sectional study conducted in the Amhara region of Northwest Ethiopia (14) in referral hospitals on precancerous lesions of the cervix among women infected with HIV found that these women were approximately 4.04 times more likely to have a precancerous lesion of the cervix than those without a history of STD.

Another cross-sectional study conducted in Saint Peter’s Specialized Hospital and a systematic review and meta-analysis in Sub-Saharan Africa indicated that those with a history of sexually transmitted infection were 1.92 times more likely to develop precancerous cervical lesions than women without such history, and having a history of STI had a significant association with precancerous cervical lesions, possibly because of the sexually transmitted nature of HPV infection. Co-infection with STI such as herpes simplex, *Chlamydia trachomatis*, or genital warts in the presence of HPV enhanced the risk of CIN by causing inflammation, which permits HPV to cause cervical lesions and carcinogenesis, and in a meta-analysis study, six observational studies showed that any disorder that was responsible for the formation of ulcers, lesions, inflammation, and any other anomalies to the cervix, vagina, genitalia, and pelvis was a very serious influencing factor for the development of precancerous cervical lesions. Sexually transmitted infections play a major role in recurrent ulcerations (3, 15).

The findings of this study indicate that a history of abortion was significantly associated with the development of precancerous cervical lesions among HIV-positive clients, with those having a history of abortion being three times more likely to develop precancerous cervical lesions than their counterparts. Another similar study conducted on Nigerian women showed that cervical cancer risk factors among HIV-infected women showed that having had five or more abortions and having other vaginal wall anomalies were related to cervical lesions or invasive cancer (16), while other systematic review and meta-analysis reports showed that from three studies, HIV-positive women with more than two births were at risk of developing precancerous cervical lesions and adaptive tissue changes (4). Similarly, another study showed that recurrent abortions were also associated with the growth of precancerous cervical lesions (16). Based on the above study results, frequent abortions create ulcerations on some of the reproductive organs, such as the uterine wall and tip of the cervix, and excessive bleeding also has a negative impact on immune suppression in women. This is associated with precancerous cervical lesions.

Different studies have shown that smoking increases the risk of precancerous cervical lesions (12, 17). Thus, this study also showed that a history of smoking had a significant association with precancerous lesions, and women with a history of smoking compared to non-smoking HIV-positive women were nine times more likely to develop precancerous cervical lesions. However, the effects of smoking on the natural history of HPV infection are not a focus, especially in women with HIV infection. Another systematic review and meta-analysis report of Japanese women showed that smoking was strongly associated with precancerous cervical lesion prevalence at baseline among HIV-infected women and was significantly associated with type 18 HPV. The general likelihood of acquiring persistent HPV was higher in smokers because of greater incidence (12, 18). A similar study conducted in South Brazilian women showed that HPV was associated with parity ≥ 3, hormonal contraceptive use, and current smoking status (19). One systematic review report indicated that 14 studies used CIN and cervical cancer as the outcome measures. Nine of these studies observed a relationship between smoking and CIN2+ (i.e., CIN2, CIN3, and cervical cancer), and five studies indicated an association between smoking and the development of CIN3+ (i.e., CIN3 and cervical cancer) (17). In this study, we concluded that smoking and HIV infection can aggravate the transmission rate of HPV infection and increase the development of precancerous and cancerous cervical lesions due to immune suppression.

### Limitations of the study

The findings of this study may not be fully generalizable to all HIV-positive women and a broader population of HIV-positive women across Ethiopia since the research was conducted in a single urban hospital.

Cross-sectional studies are limited by their ability to draw valid conclusions about the association between risk factors and health outcomes.

## Conclusions

In this study, the prevalence of precancerous cervical lesions was high among HIV-infected women in the study area. A history of abortion, smoking, and sexually transmitted infections were strongly associated with precancerous cervical lesions among women with human immunodeficiency virus.

The high proportion of precancerous cervical lesions among HIV-positive women identified in this study underscores a critical public health concern. This finding highlights the urgent need to integrate cervical cancer screening into routine HIV care. Clinically, the presence of a large number of positive cases suggests the need for immediate action to strengthen early detection and intervention. Facilities must adopt systematic screening protocols, especially for HIV-positive women, and ensure the availability of trained providers, screening equipment, and dedicated spaces to prevent service interruptions. It is also essential to address modifiable health risk factors, particularly tobacco use, and integrate family planning services to enhance women’s access and utilization.

## Recommendations

Based on the findings of this study, the prevalence of the precancerous cervical lesions was high, so we recommend the following points to institutions and concerned bodies.

Zewditu Memorial Hospital:

- The hospital should design appropriate strategies to help HIV-positive women acquire adequate services, such as expanding the existing service of family planning, which is crucial to ensuring that all HIV-positive women have access to the service, which is used for the reduction of unwanted pregnancy to tackle repeated abortion.
- The hospital should take steps to prevent the spread of STDs by strengthening counseling and treatment services for HIV-positive women.
- Smoking among young HIV-positive women could have a serious impact on cervical cancer incidence; therefore, awareness must be raised about its effect at the community level, and it is essential to pay special attention to the cessation of smoking.

Policymakers and programmers:

- Updating the integration of cervical cancer screening as a standard component of HIV care in all ART clinics requires an evaluation of service utilization.
- Increase the number of trained clinicians and designate dedicated workers for cervical cancer screening to avoid service gaps.
- Expand the screening infrastructure, including equipment and private examination rooms, to accommodate a large volume of cases.
- Increase community awareness and education initiatives to better understand the dangers of cervical cancer, especially among HIV-positive women.

Researchers:

- Conduct more operational research to investigate effective models for scaling up screening and follow-up care, program assessment, and service adoption in various contexts in Ethiopia.
- Future research should include a rural area involving multiple hospitals or regions to enhance the generalizability and applicability of the results at the national level.

## Data Availability

The raw data supporting the conclusions of this article will be made available by the authors, without undue reservation.
